# Severe homozygous HbE disease: the first case report from Nepal

**DOI:** 10.1093/omcr/omad062

**Published:** 2023-06-26

**Authors:** Urza Bhattarai, Dibasha Adhikari, Arun Gautam, Ayush Anand, Bhupendra Shah, Sanjib Kumar Sharma

**Affiliations:** Department of Internal Medicine, B.P. Koirala Institute of Health Sciences, Dharan, Nepal; Department of Internal Medicine, B.P. Koirala Institute of Health Sciences, Dharan, Nepal; Department of Internal Medicine, B.P. Koirala Institute of Health Sciences, Dharan, Nepal; B.P. Koirala Institute of Health Sciences, Dharan 56700, Nepal; Department of Internal Medicine, B.P. Koirala Institute of Health Sciences, Dharan, Nepal; Department of Internal Medicine, B.P. Koirala Institute of Health Sciences, Dharan, Nepal

## Abstract

Hemoglobin E (HbE) is the most prevalent hemoglobinopathy in the eastern Indian subcontinent. We presented the case of a 53-year-old male from Nepal with a history of multiple blood transfusions who presented with abdominal fullness for 15 years and easy fatigability for 2 months. He had pallor and massive splenomegaly. Laboratory parameters showed pancytopenia with microcytic anemia, indirect hyperbilirubinemia, target cells in the peripheral smear and iron overload. A computed tomography scan of the abdomen showed multiple splenic infarcts. Hemoglobin electrophoresis was suggestive of HbE homozygous disease. Based on these findings, we made a diagnosis of HbE homozygous disease. We provided symptomatic treatment and folic acid supplementation and counseled him for splenectomy and genetic screening. Our case highlighted the uncommon presentation of Hb E disease.

## INTRODUCTION

Hemoglobin E (HbE) is the most prevalent hemoglobinopathy in the eastern part of the Indian subcontinent and the world’s second most common hemoglobin disorder [1]. HbE homozygotes or HbE disease usually present with mild hypochromic microcytic anemia, target cells on the peripheral blood smear, and slightly increased levels of fetal hemoglobin (Hb F) [2, 3]. In addition, there is reduced survival of red blood cells with decreased osmotic fragility test [2, 3]. Rarely can the patients have splenomegaly, hepatomegaly, cholelithiasis and hyperbilirubinemia [3]. The diagnosis can be successfully made by hemoglobin electrophoresis in combination with clinical evaluation, particularly in settings where DNA testing is unavailable [2, 3]. Though most cases of HbE disease are asymptomatic or of mild severity, rarely, there can be severe manifestations [3]. Herein, we report the first case of severe HbE disease from Nepal requiring splenectomy.

## CASE REPORT

A 53-year-old male farmer , a resident of Morang in eastern Nepal, was diagnosed with abdominal fullness for 15 years, which increased since last week, and easy fatigability for two months. He underwent multiple blood transfusions in the past 5 years, with the previous blood transfusion 20 weeks ago. His family history revealed a history of multiple blood transfusions and traumatic splenic injury to his younger brother after an accidental injury by a cricket ball. There was no history of consanguineous marriage in his family and no history of any drug allergies.

On examination, his vitals were stable, and he had pallor. Abdominal examination revealed a palpable spleen 9 cm below the left costal margin, suggesting splenomegaly. There was no lymphadenopathy or hepatomegaly. The rest of the systemic examinations were unremarkable.

His laboratory investigations showed pancytopenia, unconjugated hyperbilirubinemia and decreased reticulocyte count (Table 1). Direct Coombs test and ANA were done to look for any autoimmune conditions, which were negative. A peripheral blood smear suggested a hemolytic picture (Table 1). We then performed a bone marrow aspiration and biopsy to rule out hematological malignancies. The bone marrow report showed grade 4 iron stores on Perl’s stain with a possibility of hemoglobinopathy. We performed an osmotic fragility test which was decreased. Based on these findings, we made a provisional diagnosis of hemoglobinopathy.

**Table 1:** Laboratory findings and peripheral blood smear findings of the patient

**Lab parameters**	**Value**	**Reference range**
**Hb (g/dl)**	6.7	11–16
**TLC (cells/mm** ^**3**^**)**	3100	4000–11 000
**Platelets (cells/mm** ^**3**^**)**	1,05000	150 000-450 000
**Corrected reticulocyte count**	1.3%	0.5–1.5% of total red blood cells
**MCV (fl)**	60.6	76–96
**MCH (pg)**	19.6	27–32
**MCHC (%)**	32.3	30–35
**Serum iron (μg/dl)**	197	75–175
**UIBC (μg/dl)**	4.27	125–345
**Transferrin saturation**	98%	30–40%
**Ferritin (μg/l)**	697	30–400
**TB (mg/dl)**	3.7	0.2–1.2
**UCB (mg/dl)**	3.0	0.2–1.0
**Serum vitamin B12 (ng/dl)**	314	160–1000
**Serum urea (mg%)**	24	20–40
**Serum creatinine (mg%)**	0.71	0.4–1.4
**Serum sodium (mmol/L)**	143	135–155
**Serum potassium (mmol/L)**	4.5	3.5–5.5
**LFT**		
Total protein (g/dl)	7.50	6.0–8.3
Serum albumin (g/dl)	4.84	3.5–5.0
ALT	46.94	9–43
AST	39.48	10–35
ALP	91.44	35–130
HBsAg	Negative	
Anti-HCV	Negative	
**LDH (U/L)**	270	140–280
Peripheral blood smear: Anisopoikilocytosis with microcytes, macrocytes, target cells, tear drop cells and a few spherocytes.		

Hb: hemoglobin; TLC: total leucocyte count; RBG: random blood glucose; MCV: mean corpuscular volume; MCH: mean corpuscular hemoglobin; MCHC: mean corpuscular hemoglobin concentration; UIBC: universal iron binding capacity; LDH: lactate dehydrogenase; TB: total bilirubin; UCB: unconjugated bilirubin; ALT: alanine transaminase; AST: alanine transaminase; ALP: alkaline phosphatase; LFT: liver function test; HBsAg: Hepatitis B surface antigen; Anti-HCV: antibody for hepatitis C virus.

**Figure 1:**
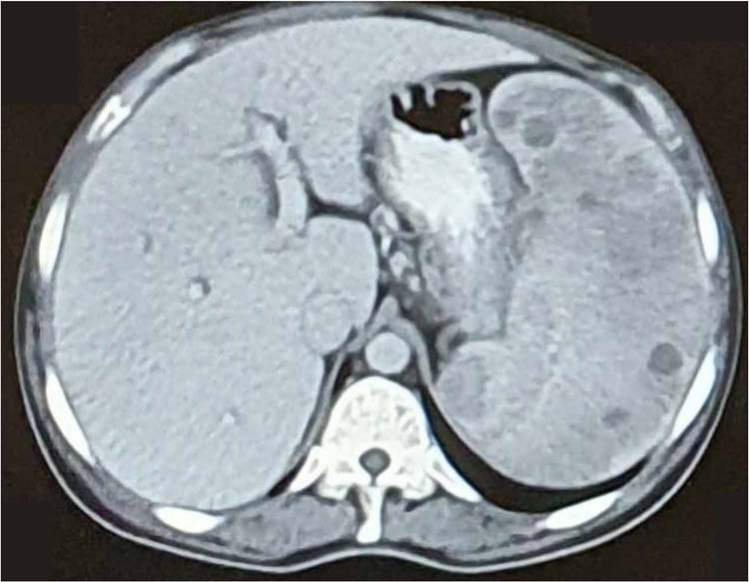
Computed tomography scan of the abdomen shows multiple hypointensities involving the spleen, most likely splenic infarcts, with no evidence of lymphadenopathy.

An ultrasound abdomen was done for abdominal distension, which showed massive splenomegaly with multiple well-defined echogenic lesions throughout the splenic parenchyma. However, no features suggested vesicular lithiasis or signs of portal hypertension. Contrast-enhanced computed tomography (CECT) of the abdomen (Fig. 1) revealed multiple hypointense lesions in the spleen, most likely splenic infarcts, without evidence of lymphadenopathy. A hemoglobin electrophoresis test was ordered to determine the type of hemoglobinopathy. The electrophoresis test using high-pressure liquid chromatography (HPLC) showed 79% HbA2 + E (Table 2). Further evaluation with capillary electrophoresis was done, which revealed a band at the HbE zone.

**Table 2:** Hemoglobin electrophoresis test

**Parameters**	**Result**	**Reference**
HbF (%)	9.50	<1.50
Hb adult (%)	6.50	83.24–90.79
Hb A2 + E (%)	79	2–3.3

Hb: hemoglobin; Hb F: fetal hemoglobin

Based on detailed investigations, we diagnosed HbE homozygous disease in severe form, presenting with multiple splenic infarcts with secondary hemochromatosis. We supplemented the patient with folic acid. Also, we counseled the patient for splenectomy and genetic screening. However, the patient refused for splenectomy.

## DISCUSSION

HbE homozygous disease is usually a mild disease without hepatosplenomegaly and requirement for blood transfusion [4, 5]. Diagnosing these patients requires detailed clinical evaluation and laboratory, radiological and genetic testing. Clinical history and examination focusing on family history, consanguinity and history of multiple blood transfusions are crucial [2]. In limited resource settings, genetic testing may be unavailable. In these settings, red blood cell indices, reticulocyte count, hemoglobin level and hemoglobin electrophoresis can aid in diagnosis [3, 4]. The typical findings include mild hypochromic microcytic anemia and mild elevation of HbF on hemoglobin electrophoresis [2, 4]. Our patient had pancytopenia with massive splenomegaly. These patients need to be evaluated systematically. A decreased hemoglobin level, microcytic picture with target cells on peripheral smear and indirect hyperbilirubinemia in our patient pointed toward hemolytic causes. However, the reduced reticulocyte count could be due to concomitant iron overload leading to bone marrow suppression [6]. Decreased osmotic fragility test ruled out hereditary spherocytosis [3]. In our patient, HPLC revealed 79% HbA2 + E. In these cases, capillary electrophoresis can quantify HbE and HbA2 [7]. In our case, capillary electrophoreses revealed the HbE band. These findings established the diagnosis of HbE homozygous disease.

In contrast to the general understanding, severe forms of HbE disease have been reported. Jayasree et al. analyzed data from 84 patients with homozygous HbE disease and found that these patients can present with variable severity, unlike the common understanding [2–5]. They found that approximately one-third of patients had splenomegaly, and 28.0% of patients had hyperbilirubinemia. However, in our case, the patient had massive splenomegaly, severe anemia, elevation of HbF and Hb A2 + E, a history of multiple blood transfusions, a hemolytic picture on the peripheral blood smear and CT findings suggestive of splenic infarcts. Severe hemolytic anemia can lead to hypoxia, and hypersplenism can lead to increased oxygen demand by the spleen. Hypersplenism in the setting of decreased oxygen due to hemolytic anemia may have led to splenic infarction in our patient [2, 8]. Our patient’s findings pointed toward a rare occurrence of a severe form of HbE homozygous disease. In addition, the bone marrow biopsy and iron profile were suggestive of secondary hemochromatosis, which resulted from iron overload due to blood transfusion. As the patient had splenomegaly, leucopenia and thrombocytopenia, we counseled the patient for splenectomy [9]. However, the patient refused for splenectomy. In addition, the allograft option was not considered as the patient belonged to a low socioeconomic status.

## CONCLUSION

HbE homozygous disease in severe form can rarely present with hemolytic anemia and massive splenomegaly.

## Data Availability

All data pertaining to this case is available within the manuscript.
